# Dyspeptic Economy

**Published:** 1871-10

**Authors:** 


### DYSPEPTIC ECONOMY.

Many a man has eaten a morsel from his plate when his appetite did not crave it, simply to prevent its being wasted. This is worse than waste.

The following is akin to a man's eating so little as to cause dyspepsia, if not a wasting and fatal disease.

" In New York State is a *very economical* woman.

" ' Waste not, want not,' is her theory. Her practice is, use up everything; so she re-fries her fries, and re-boils her boils, and re-cooks all that has been cooked. She sometimes cooks her cold potatoes four or five times; and, when refused by her family, she eats them herself, as it ' grieves her to waste anything.'

" If she makes a cake, instead of following the recipe and using four eggs, she uses one egg for two cakes; instead of using enough yeast to make light bread, she uses more saleratus, and carbonate of soda, to produce the effect, and with no success of course.

" She buys the cheapest butter, sugar, tea, flour especially, and generally the cheapest of everything — she must economize When good butter costs forty cents, she will buy that for thirty because ' she saves ten cents.'

" What, in short, is the result? Her family are dyspeptics. One has an acid stomach; another the heartburn; another poor appetite; she, herself, has medicated herself for thirty years, because she is a wretched dyspeptic.

"Yet her son takes the JOURNAL, but she does not follow its teachings.

"She dislikes to spend money for papers even — she has no money to throw away on such trash. Her hobby is to hoard money at the expense of a bad system. 'She can't afford a doctor — doctor bills are so heavy:' yet she buys every week of her life a quantity of rhubarb, peppermint, brandy, and other medicines."

By which statements our subscriber admits he has a wife whom he cannot manage; but he should take consolation in the fact that he has plenty of company. If he will only let her alone she will soon kill herself off, and then he will have another chance of having good old courting days over again. Why, the very thought of it makes a fellow's "mouth fairly water."

But no doubt this same wife is such a good one in all other respects, that he is greatly troubled at the thought of having to part with her.

